# The Role of Dyads in Subjective Reporting and Prediction of Cognitive Worsening in Cognitively Unimpaired Individuals and Individuals with Subjective Cognitive Decline: Results of the CompAS Longitudinal Study

**DOI:** 10.1093/arclin/acaf114

**Published:** 2025-12-15

**Authors:** Lucía Pérez-Blanco, Ana Nieto-Vieites, Alba Felpete-López, Sabela C Mallo, Sonali Arora, Cristina Lojo-Seoane, Onésimo Juncos-Rabadán, Arturo X Pereiro

**Affiliations:** Department of Developmental Psychology, Faculty of Psychology, Universidade de Santiago de Compostela, Santiago de Compostela, Galicia, Spain; Applied Cognitive Neuroscience and Psychogerontology group, Health Research Institute of Santiago (IDIS), Universidade de Santiago de Compostela, Santiago de Compostela, Galicia, Spain; Instituto de Psicoloxía (IPsiUS), Universidade de Santiago de Compostela, Spain; Department of Developmental Psychology, Faculty of Psychology, Universidade de Santiago de Compostela, Santiago de Compostela, Galicia, Spain; Applied Cognitive Neuroscience and Psychogerontology group, Health Research Institute of Santiago (IDIS), Universidade de Santiago de Compostela, Santiago de Compostela, Galicia, Spain; Applied Cognitive Neuroscience and Psychogerontology group, Health Research Institute of Santiago (IDIS), Universidade de Santiago de Compostela, Santiago de Compostela, Galicia, Spain; Instituto de Psicoloxía (IPsiUS), Universidade de Santiago de Compostela, Spain; Department of Developmental Psychology, Faculty of Psychology, Universidade de Santiago de Compostela, Santiago de Compostela, Galicia, Spain; Applied Cognitive Neuroscience and Psychogerontology group, Health Research Institute of Santiago (IDIS), Universidade de Santiago de Compostela, Santiago de Compostela, Galicia, Spain; Instituto de Psicoloxía (IPsiUS), Universidade de Santiago de Compostela, Spain; Department of Developmental Psychology, Faculty of Psychology, Universidade de Santiago de Compostela, Santiago de Compostela, Galicia, Spain; Applied Cognitive Neuroscience and Psychogerontology group, Health Research Institute of Santiago (IDIS), Universidade de Santiago de Compostela, Santiago de Compostela, Galicia, Spain; Instituto de Psicoloxía (IPsiUS), Universidade de Santiago de Compostela, Spain; Department of Developmental Psychology, Faculty of Psychology, Universidade de Santiago de Compostela, Santiago de Compostela, Galicia, Spain; Applied Cognitive Neuroscience and Psychogerontology group, Health Research Institute of Santiago (IDIS), Universidade de Santiago de Compostela, Santiago de Compostela, Galicia, Spain; Instituto de Psicoloxía (IPsiUS), Universidade de Santiago de Compostela, Spain; Department of Developmental Psychology, Faculty of Psychology, Universidade de Santiago de Compostela, Santiago de Compostela, Galicia, Spain; Applied Cognitive Neuroscience and Psychogerontology group, Health Research Institute of Santiago (IDIS), Universidade de Santiago de Compostela, Santiago de Compostela, Galicia, Spain; Department of Developmental Psychology, Faculty of Psychology, Universidade de Santiago de Compostela, Santiago de Compostela, Galicia, Spain; Applied Cognitive Neuroscience and Psychogerontology group, Health Research Institute of Santiago (IDIS), Universidade de Santiago de Compostela, Santiago de Compostela, Galicia, Spain; Instituto de Psicoloxía (IPsiUS), Universidade de Santiago de Compostela, Spain

**Keywords:** Subjective cognitive complaints, Dyad agreement, Informant, Hypernosognosia, Subjective cognitive decline, Longitudinal, Survival

## Abstract

**Objective:**

The main aim was to examine the value of agreement on subjective cognitive complaints (SCCs) reported by study participants and informants in predicting worsening cognitive function over time in cognitively unimpaired (CU) and subjective cognitive decline (SCD) participants.

**Methods:**

The sample consisted of 175 participants from the CompAS study (CU = 139; SCD = 36), who were followed up three times along a period from 17 to 76 months after the start of the study. Levels of agreement on the “Dyadic SCCs” were categorized according to whether informant and participant scores at baseline on the short version of the “Questionnaire d’Autoevaluation de la Memoire” were above or below a cut-off point accounting for age-related normative complaints. Two categories of agreement were identified: (a) participant scores above the cut-off and informant scores below the cut-off (“Self-over-reporting”); (b) both participant and informant scores above the cut-off point (“Agreement on presence”). We performed Cox proportional hazards regression model adjusted for sex, age, and years of education.

**Results:**

The tested model yielded statistical significant findings and acceptable model fit parameters. “Dyadic SCCs” significantly predicted cognitive worsening over time, with “Self-over-reporting” acting as a better indicator of the risk than “Agreement on presence” in both CU and SCD groups.

**Conclusions:**

The data showed that the “Self-overreporting,” compared to “Agreement on presence,” increases the risk of worsening per time unit. The findings may be explained by greater awareness of one’s own difficulties (hypernosognosia) in preclinical stages of cognitive decline.

## INTRODUCTION

### Subjective Cognitive Complaints. Clinical Relevance

Subjective cognitive complaints (SCCs) are self-expressed or informant-reported concerns about cognitive difficulties, which can range from normative age-related changes to early stages of dementia (Canevelli et al., 2013; Jack Jr. et al., 2018; Jessen et al., 2020). Subjective cognitive decline (SCD), characterized by a self-experienced persistent cognitive decline unrelated to an acute event and without objective cognitive impairment, has been proposed as an early preclinical stage of dementia (Jessen et al., 2020). Although complaints from a patient regarding a change in cognition constitute a key criterion for diagnosis of SCD (Jessen et al., 2020) and mild cognitive impairment (MCI) (Albert et al., 2011), informant-reported cognitive complaints have traditionally been considered complementary information at prodromal assessment or as a factor that simply indicates a greater likelihood of the presence of cognitive decline (Jessen et al., 2020). However, recent evidence suggests that informant reports, particularly those from close partners (Nuño et al., 2019) (i.e., spouses), may increase the accuracy of self-reports as a risk factor for dementia, from the late preclinical stages to prodromal dementia (Liu et al., 2024; Numbers et al., 2023; Pérez-Blanco et al., 2024; Truong et al., 2022).

### Dyadic Cognitive Reports

A recent narrative review by the SCD Professional Interest Area of the International Society to Advance Research and Development of Alzheimer's Treatment (ISTAART) within the Alzheimer’s Association (Nosheny et al., 2022) has pointed out the critical importance of studying dyadic cognitive reports and their validity in predicting cognitive impairment along the dementia continuum. The level of agreement or discrepancy between SCCs reported by the participant and the informant is a key aspect in this respect, providing information about the participant’s awareness of cognitive decline (ACD) (Cacciamani et al., 2020; Ryu et al., 2020; Vannini et al., 2020).

The term ACD has been coined to refer to the capacity of becoming the object of one’s own awareness about cognitive difficulties (Cappa et al., 2024). Self-awareness of the disease can vary along the continuum of cognitive decline from normative aging through preclinical stages to dementia. As a result, numerous studies have emphasized that informant reports and participant reports should be compared (Clare et al., 2011; Ryu et al., 2020; Truong et al., 2022). Three main procedures are used to assess ACD (Clare et al., 2011): (a) considering results of interviews with clinical professionals (i.e., observation of behavioral and/or verbal responses from the patient); (b) comparing self-reported SCCs and objective cognitive scores obtained in neuropsychological assessments; and (c) comparing self-reported and informant-reported SCCs measured using the same questionnaire or specific memory-related questions.

In a meta-analytical study, Cacciamani and coworkers (2021) defined two distinct patterns of awareness according to subjective reports along the continuum of cognitive decline, in individuals with and without Alzheimer’s disease (AD) pathology: (a) hypernosognosia, which occurs when older adults report more subclinical cognitive changes than their study partners (self > informant) in early preclinical stages of AD, despite objective cognitive deficits not being detected with neuropsychological tests; and (b) anosognosia, which occurs when patients declare fewer cognitive difficulties than estimated by their study partners (informant > self) in the prodromal phase (MCI) and at dementia onset, which are stages of the continuum defined by objective deterioration. The authors conclude that ACD decreases significantly from the preclinical to prodromic and dementia stages of the disease and that hypernosognosia is more prevalent in cognitively unimpaired (CU) participants. This review also noted that 72% of studies considered the subject-informant discrepancy when assessing ACD, under the assumption that the informant’s report is an accurate source of information (Cacciamani et al., 2021).

### Agreement and Discrepancy in Dyadic Reports

Evidence suggests that dyad discrepancy, especially low ACD by patient, may serve as a preclinical marker of risk for cognitive impairment (Cacciamani et al., 2017; Mimmack et al., 2023). A group of researchers from the *Institut de la Mémoire et de la Maladie d’Alzheimer* conducted the first cross-sectional study wherein low ACD (self<informant) or high ACD (self>informant) was based on the discrepancy between participants and informants, considering scores above or below reference levels corresponding to high or low percentiles in a SCD questionnaire (Cacciamani et al., 2017). This study showed that low ACD was associated with higher Aβ load and lower cortical metabolism than in individuals with high ACD. Mimmack and coworkers (2023) investigated the degree of unawareness in participants with a Clinical Dementia Rating score of 0, considering several computing procedures based on subtracting informant- and self-reports on the memory domain of the E-Cog questionnaire. This study showed that low ACD for memory decline was strongly associated with future clinical progression to AD, even after controlling for age, sex, and years of education. Notably, individuals who later experienced progression were, on average, followed up for longer than those who remained stable. This highlights the importance of long follow-up periods in detecting early changes related to awareness (Mimmack et al., 2023).

However, other studies suggested that high ACD—also referred to as the hypernosognosia phenomenon—may be associated with higher (López-Martos et al., 2024; Vannini et al., 2017) or lower (Cacciamani et al., 2022) amyloid-beta levels and could also be considered a marker in early preclinical stages before the disease progresses. Cacciamani and coworkers (2022) showed that high ACD (self > informant) was higher in CU participants (independent of the presence of positive biomarkers), whereas good agreement ACD (patient = informant) was higher in MCI participants. However, some authors have proposed agreement, either on dichotomous memory question (Gifford et al., 2014; Qi et al., 2018) or in scores above specific cut-off points in a cognitive complaint questionnaire (Gruters et al., 2019), as a predictor of future objective cognitive decline in participants without objective cognitive difficulties. Discrepant findings may be a due to the following: (a) the use of different strategies for assessing SCCs and, by extension, ACD; (b) variations in research environments (i.e., clinical settings, volunteer samples, population-based cohorts); (c) differences in focus regarding the various diagnostic groups considered (i.e., control, SCD or MCI), and (d) the influence of social and personal factors of dyadic relationship (Cacciamani et al., 2021; Nosheny et al., 2022).

### Objective

The main aim of this study was to examine the value of the agreement on SCCs reported by both participants and informants in predicting the probability that worsening of cognitive function has occurred over a period of 17–72 months in CU participants and participants with SCD. The levels of agreement between participants and informants were based on scores above or below the reference scores corresponding to the cut-off point on the SCCs questionnaire, resulting in two categories being well represented in the sample: “Agreement on presence” and “Self-over-reporting.” Considering that SCCs are common in normative aging (Mendonça et al., 2016), we established a threshold that enabled differentiation between participants undergoing normative aging and those with pre-symptomatic and prodromal stages of dementia (Pereiro et al., 2021).

## MATERIALS AND METHODS

### Study Design and Participants

This study used data from the second cohort of the Compostela Aging Study (CompAS), an ongoing longitudinal project for the early detection of cognitive decline in people aged 50 years and over in the health area of Santiago de Compostela in the autonomous region of Galicia (north-western Spain). The CompAS study began in 2016, and all subjects were followed up three times along a period from 17 to 76 months after the start of the study (*M* = 47.29; *SD* = 19.45). Data from 179 participants and their informants were included at baseline and follow-up. The following exclusion criteria were applied: previous diagnosis of neurological or psychiatric disease, specifically dementia; clinical stroke or severe cardiovascular disease; previous chemotherapy or cancer treatment; any physical or sensory condition preventing completion of assessment; history of brain damage or brain surgery; uncontrolled type II diabetes mellitus; and substance (alcohol or drug) abuse/dependency. The final sample consisted of 175 participants, after excluding four participants who were categorized in marginal categories not used in the study (i.e., “Informant-over-reporting,” “Agreement on absence”).

This research project was approved by the Galician Ethics Committee for Clinical Research (Xunta de Galicia, Spain) and was performed under the ethical standards established in the WMA Declaration of Helsinki (2008), as revised in Fortaleza 2013, the International Conference on Harmonization Tripartite Guidelines for Good Clinical Practice 1996, and the Rules Governing Medicinal Products in the European Community (Directive 91/507/EEC). All participants provided written informed consent, and anonymity was preserved.

### Neuropsychological Assessment

Comprehensive neuropsychological and cognitive assessments were administered at baseline and follow-up. An ad hoc questionnaire was used to collect sociodemographic data (e.g., age, biological sex, years of education) on participants and their informants and information on the type of relationship. The Spanish version of the Geriatric Depression Scale (Sheikh & Yesavage, 1986; Spanish version by Martínez de la Iglesia et al., 2002) was used to assess the presence of depressive symptoms, and the Charlson Comorbidity Index (Charlson et al., 1987) was used to evaluate the physical health status of participants.

Cognitive domains of interest for this study were assessed as follows: (a) Memory was assessed with the California Verbal Learning Test (Delis et al., 1987), Spanish norms by Benedet and Alejandre (1998) and the Memory Cambridge Screening Test Revised (CAMCOG-R) subscale (Roth et al., 1986) (Spanish adaptation, López-Pousa, 2003); normative score for age and education in healthy Spanish population (Pereiro et al., 2015); (b) Attention was assessed with the Trail Making Test A (Reitan, 1958) (Spanish norms NEURONORMA, Peña-Casanova et al., 2009) and the Attention and Calculation subscale of CAMCOG-R (Roth et al., 1986), Spanish adaptation (López-Pousa, 2003); normative score for age and education in healthy Spanish population (Pereiro et al., 2015); (c) Executive function was assessed with the Trail Making Test B (Reitan, 1958; Spanish norms, NEURONORMA: Peña-Casanova et al., 2009) and the Phonological fluency test (Lezak et al., 2004) through number of words initiated by the letter p in 60 s (Spanish norms, NEURONORMA: Peña-Casanova et al., 2009), and the Executive function subscale of CAMCOG-R (López-Pousa, 2003; Pereiro et al., 2015; Roth et al., 1986), and (d) language was assessed with the Boston Naming Test (García-Albea et al., 2005); Spanish norms, NEURONORMA: Peña-Casanova et al., 2009), Semantic Fluency test (animals) (Lezak et al., 2004; Spanish norms, NEURONORMA (Peña-Casanova et al., 2009) and the Language subscale of CAMCOG-R (López-Pousa, 2003; Pereiro et al., 2015; Roth et al., 1986). Functional status was also assessed with the “Lawton and Brody’s Scale for Instrumental Activities of Daily Living” (Lawton & Brody, 1969; Vergara et al., 2012).

### Measuring the Dyadic Patterns of Subjective Reports

Subjective cognitive complaints were assessed at baseline using a short seven-item Spanish version of the *Questionnaire d’auto-évaluation de la Mémoire* (QAM) (Benedet & Seisdedos, 1996; Van der Linden et al., 1989) for both participants and their informants. The QAM items were as follows: (a) “How often do you forget where you left your things?”; (b) “How often do you forget the names of people you just met?”; (c) “How often do you forget the names of close relatives or friends?”; (d) “How often do you have a word on the tip of your tongue?”; (e) “How often do you find yourself lost in familiar places where you have been before?”; (f) “How often do you find yourself lost in unfamiliar places where you have been a few times?”; and (g) “How often do you forget things you planned to do?.” The total score for subjective reports of dyads was obtained using a five-point Likert scale (with 1 indicating “never” and 5 indicating “always”) and ranged from 7 to 35.

Identification of the cut-off point was further established using the fifth percentile of the QAM, which is a valid measure of SCCs severity for predicting progression to MCI and dementia (Pereiro et al., 2021). The “Dyadic SCCs” measure was computed considering the severity of informant and participant QAM scores above or below a cut-off point (i.e., 10–11). “Dyadic SCCs” were categorized as follows: (a) Agreement on the absence of SCCs (“Agreement on absence”), when QAM total scores of both informant and participant were below the established cut-off point—both QAM total scores ≤10–; (b) Agreement on the presence of SCCs (“Agreement on presence”), when QAM total scores of both informant and participant are above the established cut-off point—both QAM total scores ≥11–; (c) Over-reporting by participants (“Self-over-reporting”), when QAM total scores are above the established cut-off point in patient and below in the informant—QAM patient-score ≥11 and QAM informant-score ≤10; and (d) Over-reporting by informants (“Informant-over-reporting”) when QAM total scores are above the established cut-off point in informant and below in the participant—QAM patient-score ≤ 10 and QAM informant-score ≥11.

### Clinical Diagnoses

At the consensus diagnosis meeting, the participants were classified in the following categories: CU, SCD, MCI, or major neurocognitive disorder (or dementia).

A diagnosis of CU was made when participants did not meet the criteria for objective cognitive impairment, tested by the Spanish version (López-Pousa, 2003) of the Cambridge Cognitive Assessment–Revised (Roth et al., 1986) battery according to age and formal education norms (Pereiro et al., 2015) and when their scores in the QAM were below the fifth percentile according to age norms (Pereiro et al., 2021).

Diagnosis of SCD was according to the criteria proposed by Jessen and coworkers (2014). First, self-expressed concern in cognitive capacity relative to a few years previously. Second, objective cognitive performance on standardized cognitive tests remained within the normal range for age and years of education (scores that do not exceed the cut-off established for the diagnosis of MCI, i.e., between 1 and 2 SDs below the mean according to published normative values), and preserved autonomy in instrumental activities of daily living (IADL). Normal cognitive status was verified by the same procedure as for the CU participants. In addition, given the presence of cognitive complaints in normative aging (Rabin et al., 2015), we considered the severity of concern (Jessen et al., 2020), and participants were only categorized in the SCD group when complaints exceeded the score in the QAM that corresponds to the fifth percentile according to age norms (Pereiro et al., 2021).

MCI was diagnosed according to the following criteria (Albert et al., 2011; American Psychiatric Association, 2013; Dubois et al., 2007; Petersen, 2004): (a) presence of cognitive complaints in the participant preferably corroborated by the informant; (b) evidence of cognitive impairment confirmed through objective testing in one or more cognitive domains (between 1 and 2 SDs below published normative data according to age and education adjusted-norms); (c) preservation of independence in IADL (Lawton and Brody Index) (Lawton & Brody, 1969; Vergara et al., 2012); and (d) non-fulfilment of diagnostic criteria for dementia.

Diagnosis of Dementia—Major Neurocognitive Disorder—was based on the Diagnostic and Statistical Manual of Mental Disorders, Fifth Edition (American Psychiatric Association, 2013) as follows: (a) evidence of cognitive impairment in one or more cognitive domains. This criterion was considered fulfilled when the scores were below the 2 SDs according to age and educated adjusted-norms; (b) having impaired IADL; (c) deficits do not appear exclusively during delirium; and (d) the alteration is not better explained by the presence of other affective disorders (e.g., Major Depressive Disorder).

At each follow-up assessment, participants diagnosed at baseline as CU (*N* = 139) and SCD (*N* = 36) were classified as follows: (a) stable, when the diagnosis at baseline remains unchanged from baseline to follow-up, and (b) worsening, when the diagnosis at baseline converted to MCI and/or dementia at some point during follow-ups. Individuals who progressed from CU to SCD were considered stable (*N* = 39; 20.6%). The pattern of clinical reversion from MCI to CU or SCD was not considered because only six cases (1.2%) were observed in the total sample. Reversion from SCD to CU also occurred at a very low level (0.8%), as participants’ baseline diagnoses were conservatively corrected from the first follow-up when the diagnosis suggested recovery (Pereiro et al., 2021).

### Statistical Analysis

All statistical tests were performed with the IBM SPSS software 29, and statistical significance was set at *p* < .05. One-way ANOVA, χ^2^, and Kruskal–Wallis tests were conducted to analyze differences in sociodemographic, physical, emotional, and cognitive variables between diagnostic groups (CU and SCD). Levene’s test examined the assumption of equal variance across groups. Analysis of covariance (ANCOVA), with age and years of education as covariates, was conducted to assess whether the performance in the neuropsychological variables differed between the groups.

Survival analyses were run using a Cox proportional hazards (PHs) regression model (with no cases censored) applying the Enter method. The event was defined as worsening cognitive status versus stability over a follow-up period of 17–76 months. The “Dyadic SCCs” with two values, “Agreement on presence” (value 0) and “Self-over-reporting” (value 1) at baseline were included in this analysis as predictor variable. The other two categories of the “Dyadic SCCs”—“Informant-over-reporting” and “Agreement on absence”—were discarded because their presence in our sample was very marginal (*N* = 2, 1.1% in each category). Age, years of education at baseline, and biological sex were also included as covariates. Two groups diagnosed at baseline (CU and SCD) were included in the model as strata. The final sample of 175 participants was included in this survival analysis (CU = 139; SCD = 36). We assessed assumption of PHs across covariates by inspecting the log-minus-log plots (Kuitunen et al., 2021) that are displayed as figures in the Results section. The Variance Inflation Factor (VIF) was used to detect multicollinearity between covariates. We also plotted as figures the shape of the survival functions for the predictors.

## RESULTS

### Descriptive Sample and Relationship Type of Dyads

The characteristics of all participants at baseline (stratified by clinical diagnosis) and informants age, sex and the participant-informant relationship are summarized in Table 1.

**Table 1:** Baseline characteristics of all participants [mean (*SD*) and min-max range] and their informants

VARIABLE	**CU** (*N* = 139)	**SCD** (*N* = 36)	***Omnibus Test***	***p***
**Sociodemographic—Participants**				
Age (in years)	63.33 (8.37), 50–84	69.61 (7.84), 50–84	^a^ *F*(1,174) =16.48	<.001^**^
Biological sex (% women)	107 (77%)	26 (72.2%)	^b^χ^2^ = 0.35	.552
Years of education	12.48 (5.68), 3–25	10.00 (4.97), 3–25	*F*(1,174) =5.72	.018^*^
**Health Status**				
Charlson Index	0.259 (0.50), 0–2	0.388 (0.64), 0–2	^c^ *H*(1) =1.11	.295
GDS-15	2.41 (2.45), 0–12	3.08 (2.01), 0–12	*F*(1,174) =2.25	.135
Lawton and Brody Scale (by informant reported)	7.79 (0.56), 5–8	7.52 (0.94), 5–8	*H*(1) =3.11	.077
**Subjective Reports**				
QAM participant-score	15.71 (2.66), 11–26	19.63 (2.31), 11–26	*F*(1,174) =65.04	<.001^**^
QAM informant-score	14.29 (3.77), 7–28	15.91 (3.38), 7–28	*F*(1,174) =5.48	.020^*^
**Sociodemographic—Informants**				
Age (in years)	56.64 (14.47), 19–85	57.83 (14.40), 18–83	*F* (1,171) =0.19	.661
Biological sex (% women)	77 (55.39%)	21 (58.33%)	χ^2^ = 0.10	.752
**Dyadic Relationship Type**				
Relationship (% spouse and/or child)	109 (78.42%)	29 (80.55%)	χ^2^ = 0.08	.779

^a^One-way ANOVA.

^b^Chi squared.

^c^Kruskal–Wallis test.

^*^ *p* < .05.

^**^ *p* < .01.

CU = cognitively unimpaired; GDS-15: Geriatric Depression Scale; QAM = Questionnaire d’auto-évaluation de la Mémoire; SCD = subjective cognitive decline. Significant *p* values in bold.

Of the total sample, the SCD participants were significantly older and had fewer years of education than the CU participants. Seventy-six percent of all participants were women (*n* = 133), and only 24% were men (*n* = 42)*.* However, the groups did not differ with respect to biological sex. There were no significant differences between the CU and SCD groups regarding health status, functional status, or depressive symptoms.

As expected, subjective complaint scores from participants and informants were significantly higher for the SCD than for CU groups. Informants from the CU and SCD groups were similar regarding the sociodemographic characteristics considered. Informants were usually spouses and/or children (85.10% vs. 71.60%; χ^2^ = 4.75; *p* = .029) for “Agreement on presence” than for “Self-over-reporting.” A significant difference in the frequency of the dyadic patterns was observed (χ^2^ = 6.24; *p* = .012), with CU participants showing a higher “Self-over-reporting” than “Agreement on presence” (51.15% vs. 48.85%), whereas the opposite pattern was observed for the SCD participants (27.78% vs. 72.22%).

Across the follow-up, 40 of the 175 participants experienced objective cognitive worsening (22.85% of the total sample: 23 from CU and 17 from SCD). These participants tended to be older (67.90 [*SD* = 8.91] vs. 63.65 [*SD* = 8.33]) years], had a lower level of education (10.05 [*SD* = 4.79] vs. 12.54 [*SD* = 5.73] years), but with no sex/gender differences. SCD participants were more likely to be diagnosed with MCI and/or dementia (47.22% vs. 16.54%) than CU participants (χ^2^ = 12.260; *p* < .001).

The results of the ANCOVA of neuropsychological scores obtained by the diagnostic groups are shown in Table 2. SCD participants performed significantly worse than CU participants on three cognitive measures, CAMCOG-R total score, CAMCOG-R Executive subscale, and Semantic Fluency.

**Table 2:** Neuropsychological profile (mean values and standard deviations) of CU and SCD groups at baseline

	**CU** (*N* = 139)	**SCD** (*N* = 36)	**ANCOVA** ^a^	***p***
**Global Cognitive Functioning**				
MMSE screening	28.64 (1.32)	28.03 (1.71)	*F*(3,171 = 0.959	.329
CAMCOG-R total score	94.63 (6.43)	89.94 (6.51)	*F*(3,171) = 4.208	.042^*^
**Memory**				
Memory-CAMCOG-R subscale	22.69 (2.47)	21.16 (2.04)	*F*(3,171) = 3.495	.063
Immediate total-CVLT	52.74 (9.17)	48.83 (11.09)	*F*(3,171) = 0.001	.974
Short-term delayed recall CVLT	10.94 (2.73)	9.69 (3.00)	*F*(3,171) = 0.068	.794
Long-term delayed recall CVLT	11.87 (2.64)	10.63 (2.58)	*F*(3,171) = 0.233	.630
**Attention and Processing Speed**				
TMT-A (in seconds)^b^	47.03 (19.95)	52.11 (19.79)	*F*(3,171) = 0.750	.388
Attention-CAMCOG-R subscale	7.79 (1.49)	7.75 (1.20)	*F*(3,171) = 0.032	.858
**Executive Functioning**				
TMT-B (in seconds)^b^	123.45 (70.57)	147.86 (70.33)	*F*(3,171) = 0.449	.504
Phonological fluency (initiated by p-letter)	14.78 (5.45)	11.86 (4.46)	*F*(3,171) = 0.950	.331
Executive-CAMCOG-R subscale	21.89 (4.06)	18.44 (4.56)	*F*(3,171) = 0.5.841	.017^*^
**Language**				
Language-CAMCOG-R subscale	27.38 (1.96)	26.00 (2.16)	*F*(3,171) = 3.615	.059
Semantic fluency (animals)	20.00 (5.53)	16.27 (4.14)	*F*(3,171) = 4.119	.044^*^
BNT-60 item	51.66 (7.21)	47.75 (6.32)	*F*(3,171) = 1.999	.159

^a^ANCOVAs were controlled for participant age and years of education.

^b^Total score > 300 s = missing/not applicable.

*Note*: Significance was set at ^*^p < .05.

BNT = Boston Naming Test; CAMCOG-R = Cambridge Screening Test Revised; CU = cognitively unimpaired; CVLT = California Verbal Learning Test; MMSE = Mini-Mental State Examination; SCD = subjective cognitive decline; TMT = Trail Making Test. Significant *p* values in bold.

### Subjective Reports of Dyads and Worsening Cognitive Function

The Cox PHs regression model was statistically significant (model fit: −2 *Log Likelihood* = 1304.684, χ^2^ = 10.82, *df* = 4, *p* = .029). “Dyadic SCCs” significantly predicted cognitive worsening (β = 0.50, *SE* = 0.16, *Wald* = 9.49, *p* = .002). The estimated risk based on the *Hazard Ratio* value was 1.65 (95% CI = 1.20–2.28). These results indicated that, in both the CU and SCD groups, participants belonging to the dyad corresponding to the “Self-over-reporting” category exhibited a 65% greater risk of objective cognitive worsening for each month since baseline compared to participants belonging to the dyad that corresponds to the “Agreement on presence.” Age, sex, and years of education were not significant factors (see Table 3).

**Table 3:** Cox regression model results on risk objective cognitive worsening

	**β**	***SE***	***Wald***	***p***	***HR***	***95.0%CI***
**VARIABLES**						
Overreporting by participants (dyadic SCCs)	0.50	0.16	9.49	.002^*^	1.65	1.20–2.28
Women (sex)	−0.09	0.18	0.27	.59	0.91	0.62–1.31
Age	0.01	0.01	0.64	.42	1.01	0.98–1.03
Years of education	−0.01	0.02	0.14	.71	0.99	0.96–1.03

*Note*: Models were adjusted for age, biological sex, and years of education.

^*^ *p* < .05.

Abbreviations: HR = hazard ratio; SCCs = subjective cognitive complaints. Significant *p* values in bold.

**Fig. 1:**
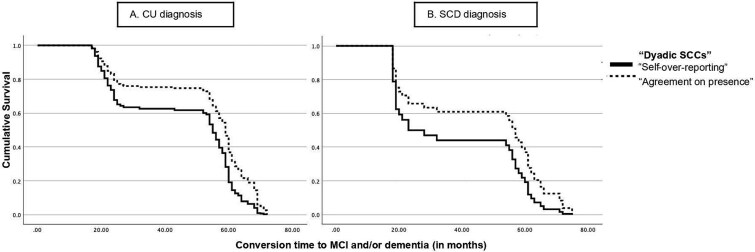
Survival function using “Dyadic SCCs” over time conversion. Legends: Black line refers to “Self-over-reporting” pattern”; black dotted line represents “Agreement on presence.” Abbreviation: SCCs = subjective cognitive complaints.

The survival function for conversion to MCI and/or dementia (cognitive worsening) for CU and SCD groups over time (in months) is displayed in Fig. 1. The cumulative survival should be interpreted in terms of risk, because the risk of cognitive worsening increases as the cumulative survival rate decreases. In Fig. 1A and B, the solid lines, which correspond to over-reporting by participants, indicate a lower cumulative survival rate than the dashed lines, which correspond to agreement. The survival functions plotted show that the probability of cognitive decline occurring earlier was greater for the SCD group (Fig. 1B) than for the CU group (Fig. 1A), and this probability is greater for dyads where significant complaints were expressed only by participants (i.e., “Self-overreporting”) than in those where there was coincidence in expressing complaints (i.e., “Agreement on presence”). The length of follow-up did not differ significantly between groups (Mann–Whitney U = 2044; *p* = .091).

**Fig. 2:**
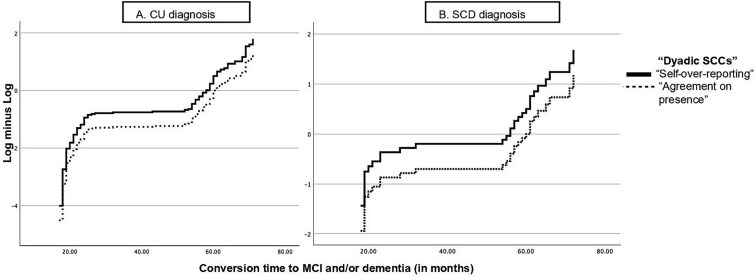
Log minus log (LML) function using “Dyadic SCCs” over time of conversion. Legends: Black line refers to “Self-over-reporting” pattern”; black dotted line represents “Agreement on presence.” Abbreviation: SCCs = subjective cognitive complaints.

The log(−log) plots shown in Fig. 2 tested the PH assumption for CU (chart 2A) and SCD (chart 2B) groups, as the main assumption for Cox analysis. In the plots, the two lines representing covariates (Self-over-reporting and Agreement on presence) are almost parallel and never intersect. VIF values for the four predictor variables range from 1.03 to 1.33, indicating the absence of multicollinearity.

## DISCUSSION

Numerous studies on the validity of self- and informant reports for predicting the risk of cognitive decline have been published (see Nosheny et al., 2022). However, very few studies have analyzed the utility of analyzing agreement and discordance between self- and informant-reported SCCs at baseline in predicting the time to cognitive worsening.

### Agreement and Discrepancy in CU and SCD Dyadic Reports

The study results showed that prevalence of “Self-over-reporting” (51.15%) was slightly higher in dyads including CU participants, whereas “Agreement on presence” (72.22%) was more frequent in dyads including SCD participants, suggesting earlier sensitivity to cognitive changes in patients than in informants and a greater ability to detect these in informants as the disease progresses. The level of cognitive complaints has previously been reported to be higher in CU participants (Ab− and Ab+) than in their informants, and self-reports (but not informant reports) were able to discriminate between CU subgroups with and without amyloid burden (Cacciamani et al., 2022). Our results showed that “Dyadic SCCs” adjusted for biological sex, age, and years of education were associated with time-to-onset of cognitive worsening. The risk of cognitive worsening was higher in participants who reported complaints in the almost complete absence of agreement from informants (“Self-over-reporting”) than in participants who agreed with informants by expressing complaints of a certain intensity (“Agreement on presence”) in both CU and SCD groups. Moreover, the survival functions show that the probability of objective cognitive decline occurring earlier in time is greater for the SCD group than for the CU group. These results support the inclusion of SCD as a presymptomatic stage in the dementia continuum (Dubois et al., 2007) and highlight the need to consider the severity of concerns to further identify the presymptomatic stage (i.e., SCD) and its associated risk at the onset of dementia (Jessen et al., 2020; Pereiro et al., 2021).

### Awareness in Presymptomatic Stages of Dementia

The scientific evidence could be interpreted from the point of view of the phenomenon of hypernosognosia proposed by Vannini et al. (2017). Some studies suggest that hypernosognosia may emerge before anosognosia in the early stages of the cognitive continuum of dementia, whereas anosognosia would appear later, during the prodromal phase of MCI (Cacciamani et al., 2021; López-Martos et al., 2024; Vannini et al., 2017). Some longitudinal evidence showed that at the beginning of the deterioration process, self-reports could contribute as much as agreement with the informant in the presence of complaints in early stages of clinical progression (Amariglio et al., 2015; Gifford et al., 2014), although informant reports considered separately can be useful in predicting late cognitive changes (Numbers et al., 2023; Pérez-Blanco et al., 2024). Our findings support the predictive value of over-reporting self-complaints in preclinical stages of dementia (Cacciamani et al., 2021; Cacciamani et al., 2022; López-Martos et al., 2024; Vannini et al., 2017), in contrast to evidence pointing out the agreement between self- and informant-report expressing cognitive complaints (Gifford et al., 2014; Gruters et al., 2019; Qi et al., 2018). The “Self-over-reporting” in our preclinical CU and SCD participants implies a greater risk of objective worsening of cognitive function over time than “Agreement on presence.” The risk, according to our survival functions, is more likely to occur earlier in the SCD group than in the CU group. Thus, confirmation by a family member seems to play a more important role in prodromal stages in the light of understanding of the participant’s ACD (Clare et al., 2011; Liu et al., 2024; Ryu et al., 2020; Truong et al., 2022) or anosognosia (Hanseeuw et al., 2020; Munro et al., 2018; Vannini et al., 2017). The fact that deterioration times are longer when informants’ and participants’ reports match may also be related to other factors such as the indirect beneficial effect of close relationships with the informants’, who are more likely to become aware of their relative’s cognitive difficulties early (Colloby et al., 2022).

In terms of the risk associated with cognitive worsening in CU and SCD participants, our study results shed new light on the role of over-reporting (self > informant) as an early cognitive predictor. This is also consistent with the findings of several studies (Hanseeuw et al., 2020; López-Martos et al., 2024), which showed that greater ACD may be particularly important for identifying people at risk of AD in both clinical and research contexts. Our findings suggest that assessing ACD by comparing self-reports and informant reports may help general practitioners make a more timely and accurate identification of “*worried-well*” patients with persistent cognitive decline but no evidence of AD pathology and cognitive impairment as undergoing normative cognitive aging with SCCs (Cacciamani et al., 2021; Jessen et al., 2020; Molinuevo et al., 2017).

### Limitations

Finally, some limitations must be considered. The discrepancy between self- and informant-report implicitly assumes that the informant is the better estimator of the participant’s cognitive changes. However, informant reporting can also be influenced by certain personal factors (e.g., personality traits, anxiety, depression) or relational factors (e.g., participant-informant relationship, frequency of social contact) not considered in this study (Aschwanden et al., 2020; Burmester et al., 2016; Crumley et al., 2014; Markova et al., 2017). Specifically, the presence of depressive symptoms should be considered in future research, due to their influence on cognitive complaints (Jessen et al., 2020) that could affect the assessment of ACD. Unfortunately, we were not able to analyze the validity of participant unawareness in predicting AD progression (Cacciamani et al., 2017; Mimmack et al., 2023) because of the lower prevalence (1.1% of the total sample) of “Informant-over-reporting” (i.e., informant > self) category in our sample. The inclusion of a larger sample size would have better clarified the presence/absence of low awareness patterns (“Informant-over-reporting”) in presymptomatic stages and their association with the risk of AD pathology. Further research on this topic is required to determine the role that the informant plays in the clinical transition of participants with early and late MCI across the dementia spectrum. We suggest, as a future hypothesis, that high awareness by informants (informant > self) may emerge in early prodromal stages of dementia, rather than in preclinical stages, where the patient’s awareness of decline is preserved.

## CONCLUSION

In summary, our findings support the importance of jointly considering dyadic subjective reports and comparing information from participants and informants to test the patient’s awareness of cognitive deficits along the continuum of cognitive decline to dementia. In this context, greater self-awareness, or “Self-over-reporting” (hypernosognosia), at preclinical stages of AD may be an early subjective sign of cognitive decline. “Self-over-reporting” could be influenced by a possible depression, making a differential diagnosis necessary.
